# Ultrasonic-assisted, additive-free Pd-catalyzed Suzuki–Miyaura cross-coupling enabled synthesis of novel arylated benzofuran-triazole hybrids

**DOI:** 10.3389/fchem.2025.1726528

**Published:** 2025-12-10

**Authors:** Muhammad Jawwad Saif, Sajjad Ahmad, Aqsa Mushtaq, Saba Munawar, Ameer Fawad Zahoor, Shoela Ettampola, Ali Irfan, Katarzyna Kotwica-Mojzych, Karol Ruszel, Mariusz Mojzych

**Affiliations:** 1 Department of Applied Chemistry, Government College University Faisalabad, Faisalabad, Pakistan; 2 Department of Chemistry, University of Engineering and Technology Lahore, Faisalabad Campus, Faisalabad, Pakistan; 3 Department of Chemistry, Government College University Faisalabad, Faisalabad, Pakistan; 4 Dunwoody High School, Dunwoody, GA, United States; 5 Department of Histology, Embryology and Cytophysiology, Medical University of Lublin, Lublin, Poland; 6 Collegium Medicum, The Mazovian Academy in Płock, Płock, Poland

**Keywords:** Suzuki reaction, Pd(PPh_3_)_4_, ultrasound, arylated benzofuran-triazole hybrids, heterocycles

## Abstract

An additive-free and ultrasound-assisted approach has been established for the Suzuki–Miyaura cross-coupling reaction between substituted boronic acid and benzofuran-endowed aryl halides by employing a catalytic amount of Pd(PPh_3_)_4_. The resulting substituted biaryls were then employed as efficient starting materials to furnish a novel library of arylated benzofuran-triazole hybrids **13(a–i)**. The method offers a facile, efficient, and less time-consuming approach under non-inert conditions to synthesize the targeted derivatives in a good to excellent yield range of 70%–92%.

## Introduction

Carbon–carbon bond-forming reactions play a crucial role in the synthesis of several intricate organic compounds (Akhtar et al., 2019; Brahmachari, 2016; Mushtaq et al., 2024; Zahoor et al., 2024a). Among several carbon–carbon (C-C) bond formation reactions, the Suzuki–Miyaura cross-coupling reaction (SMCR) has found huge significance since its discovery by Akira Suzuki and Norio Miyaura in 1979 (Miyaura et al., 1979). They carried out the reaction by treating aryl halides with alkenylboranes employing tetrakis (triphenylphosphine)palladium Pd(PPh_3_)_4_ as a catalyst (Miyaura and Suzuki, 1979). As a Suzuki–Miyaura coupling reaction involves the linkage of a Csp^2^-Csp^2^ bond, it is predominantly involved in the synthesis of planar compounds, thereby validating its efficiency in the drug development process (Miyaura and Suzuki, 1995). In Pd-catalyzed cross-coupling reactions, aryl iodides (as Suzuki coupling partners) are generally the most reactive aryl halides (Panahi et al., 2018). Generally, aryl bromides are more commonly employed than aryl chlorides, whose reactivity is less than that of aryl iodides and bromides (Miyaura and Suzuki, 1995; Sonogashira, 1998; Stanforth, 1998; Suzuki, 1999; Miyaura, 1998). However, in a few Pd-catalyzed reactions, aryl bromides involving cross-coupling reactions have been observed to proceed more rapidly than aryl iodides. Moreover, Suzuki reactions involving aryl bromides have been reported to occur more rapidly under room temperature conditions than those involving aryl iodides (Littke et al., 2000).

SMCR involves the facile synthesis of biaryl scaffolds, which are abundantly found in several pharmaceutically active and synthetically significant organic molecules (Adlington et al., 2018; Farhang et al., 2022; Kotha et al., 2002). This reaction has also found applications in the synthesis of natural products (Taheri Kal Koshvandi et al., 2018). Non-toxicity, readily accessible reagents, and high stereo- and regioselectivity are some of the merits of SMCR over other coupling reactions. The reaction proceeds by employing homogeneous catalysis, thus offering remarkable activity and selectivity (Suzuki, 1999; Suzuki, 2002; Johansson Seechurn et al., 2012; Zhang and Wang, 2015). Several Pd-based homogenous catalysts have been employed for this reaction, including Pd(II) complexes (Nagalakshmi et al., 2020; Ortega-Gaxiola et al., 2020; Thunga et al., 2019), PdCl_2_ (Nobre et al., 2018), Pd_2_dba_3_, Pd(PPh_3_)_4_ (Czompa et al., 2019; Suzuki and Yamamoto, 2011), Pd(PPh_3_)_2_Cl_2_ (Mai et al., 2019; Sarkar et al., 2020), Pd (dppf)Cl_2_ (Alza et al., 2015), and Pd(OAc)_2_ (Li et al., 2018; Louvis et al., 2016). Most of these reactions have been carried out under an inert atmosphere (nitrogen or argon) (Wang et al., 2001).

Several heterogeneous catalytic systems, which include metal-based heterogeneous catalysts trapped on water-insoluble support and palladium supported on carbon (Pd/C), have also been introduced for this reaction. These catalytic systems have been found to be favorable in terms of facile recovery of the catalyst, low catalyst requirement, and high catalytic activity (Amoroso et al., 2011; Sancheti and Gogate, 2018; Zheng et al., 2013). However, these catalysts have unveiled several deficiencies, including a need for additives, long reaction times, and more palladium content (Felpin et al., 2006; Maegawa et al., 2007; Polshettiwar et al., 2010; Rao et al., 2014). These notable shortcomings must be avoided by investigating novel methods for the Suzuki coupling reaction to carry out this significant reaction in low catalyst loading within short reaction times.

Various chemical and physical changes that occur as a result of cavitational events in an ultrasound reactor make it an effective apparatus for the facile conversion of reactants into target molecules. Ultrasound is processed through multiple rarefaction and compression cycles that are produced in a liquid medium. Upon passing through several growth stages, these cavities eventually partially collapse, leading to severe local heating and high pressure (Cintas et al., 2014; Mason, 1997). In addition, the rapid disintegration of the cavitating bubble results in liquid circulation and severe agitation. These physical effects facilitate the breakage of solid aggregates. These factors may also contribute to providing a large surface area, thereby facilitating the reaction to proceed rapidly. For these reasons, ultrasound-assisted methodologies are considered greener approaches as they involve intense processing and moderate reaction conditions with reduced probabilities of side products (Brahmachari et al., 2021; Cravotto et al., 2015; Irfan et al., 2022).

The literature survey highlights the employment of ultrasonic conditions for Suzuki–Miyaura coupling to access diverse organic compounds. The reported protocols involved the use of palladium-based catalysts, that is, Pd/C (Cravotto et al., 2005), Pd/PVP (de Souza et al., 2008), and Pd(PPh_3_)_4_ (Cella and Stefani, 2006) under ultrasonic conditions. Similarly, synthesis of polydihexylfluorene has also been carried out by using an ultrasound-assisted Suzuki cross-coupling reaction (Gao et al., 2014). All these instances emphasize the applications of ultrasound irradiation toward the fast and facile conversion of Suzuki coupling partners into desired coupling products.

**FIGURE 1:**
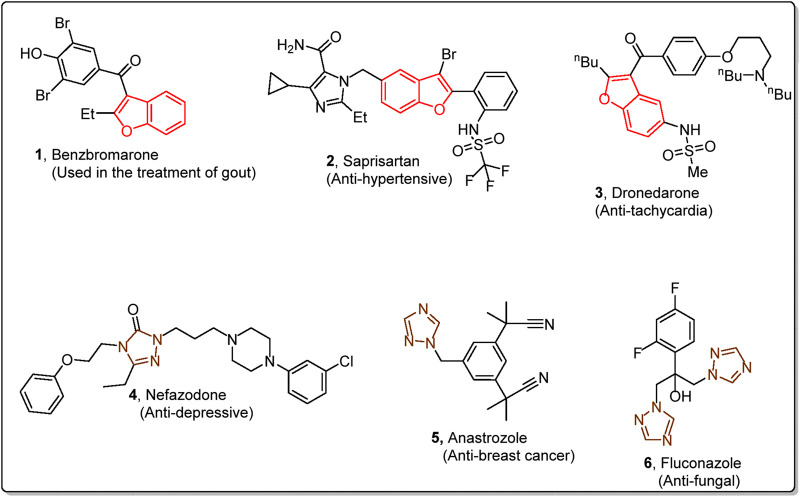
Structures of some benzofuran and triazole core-constituting drugs.

Heterocycles are integral structural constituents of several bioactive organic compounds and drugs. Benzofuran and triazole are oxygen and nitrogen-containing heterocyclic scaffolds that are key structural units of several pharmaceutically significant organic compounds and therapeutic agents (Bozorov et al., 2019; Khanam and Shamsuzzaman, 2015). Benzbromarone **1**, amiodarone **2**, and saprisartan **3** are some of the clinically established benzofuran-endowed drugs. These drugs are known to treat gout, tachycardia, and hypertension, respectively (Nevagi et al., 2015). Similarly, nefazodone **4**, anastrozole **5**, and fluconazole **6** are some triazole-bearing market-available drugs that are effective against depression, breast cancer, and fungal diseases, respectively (Figure 1) (Kharb et al., 2011).

**SCHEME 1:**
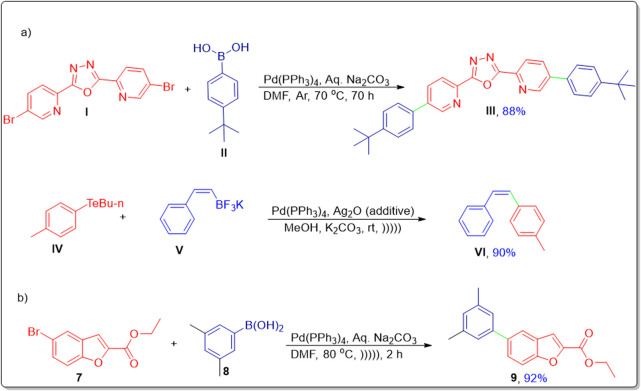
**(a)** Previous synthetic routes. **(b)** Our synthetic strategy.

Bearing in mind the significance of ultrasound-assisted approaches and pharmaceutical significance of these heterogeneous scaffolds, we have carried out the novel synthesis of arylated benzofuran-triazole hybrids by utilizing a novel additive-free approach for Suzuki–Miyaura coupling, constituting low catalyst loading of tetrakis (triphenylphosphine) palladium under ultrasonic irradiation and a non-inert atmosphere (Scheme 1).

## Results and discussion

The 5-bromo-substituted benzofuran-based ester was synthesized using the reported protocol (Faiz et al., 2019). The synthesized halogenated ester **7** was employed in a model Suzuki–Miyaura cross-coupling reaction with 3,5-dimethyl benzene boronic acid **8** using several palladium-based catalysts in the presence of different bases. The optimization of reaction conditions was carried out by varying the Pd catalyst and bases under conventional heating and ultrasonication irradiation in dimethylformamide (DMF). Initially, the Suzuki reaction was investigated using palladium (II) acetylacetone (Pd (acac)_2_) and aqueous sodium carbonate (base) under conventional conditions, which gave the resulting coupled molecule **9** in 10% yield (Table 1, entry 1). The reaction was repeated at 90 °C using potassium phosphate as a base, which was found to diminish the yield of target molecule **9** (8%). Similarly, utilization of tris(dibenzylideneacetone)dipalladium (0) (Pd_2_ (dba)_3_) as a catalyst employing different bases (aq. Na_2_CO_3_ and Cs_2_CO_3_) at 80 °C and 60 °C (respectively) afforded the target molecule **9** in 50% and 30% yield (Table 1, entries 3 & 4). It was observed that the Suzuki reaction carried out using aqueous sodium carbonate afforded a relatively better yield (50%) of the target molecule in a shorter duration (6 h) than the one obtained by using cesium carbonate, which afforded a 30% yield of product in 8 h. However, when the same reaction was carried out under ultrasonic conditions using aq. Na_2_CO_3_, 50% yield of target molecule **9** was obtained in relatively less time (4 h) (Table 1, entry 5). The catalytic effects of [1,1′-bis(diphenylphosphino)ferrocene]dichloropalladium (II) (Pd (dppf)Cl_2_) and bis(triphenylphosphine)palladium (II) dichloride (PdCl_2_(PPh_3_)_2_) were also explored by employing two different bases using conventional and ultrasonication conditions. A Pd (dppf)Cl_2_-promoted and ultrasonic-assisted Suzuki reaction involving aqueous sodium carbonate/aqueous potassium carbonate afforded target molecule **9** in 52% and 55% yields, respectively (Table 1, entries 6 and 8). A Pd (dppf)Cl_2_ catalyst in the presence of aqueous sodium carbonate and potassium carbonate (base) at 25 °C furnished the target molecule **9** in 48% and 52% yields, respectively (Table 1, entries 7 and 9). The PdCl_2_(PPh_3_)_2_-catalyzed model reaction carried out by employing sodium carbonate resulted in a relatively higher yield (60%) of the targeted product **9** than the reaction carried out in aqueous potassium carbonate (53%) (Table 1, entries 10 and 11). Repeating the same reaction under ultrasonic irradiations harnessing aq. Na_2_CO_3_ furnished a higher yield (67%) of product **9** in a relatively shorter duration of 2 h (Table 1, entry 12). The catalytic efficiency of tetrakis (triphenylphosphine)palladium (0) (Pd(PPh_3_)_4_) was also investigated under ultrasonication conditions using these two bases. It was observed that the use of 5 mol% of this catalyst in the model reaction and using aqueous sodium carbonate as a base resulted in a remarkable yield of 92% of the targeted molecule **9** (Table 1, entry 13). Meanwhile, employment of potassium carbonate within a Pd(PPh_3_)_4_-mediated model reaction diminished the yield of coupled product 9 i.e., 85%. (Table 1, entry 14), thereby indicating the use of the Pd(PPh_3_)_4_ catalyst, aq. Na_2_CO_3_, and ultrasonic irradiation as the best optimized conditions for the studied reaction.

**TABLE 1:** Optimization of reaction conditions^a^ ^/^ ^b^.

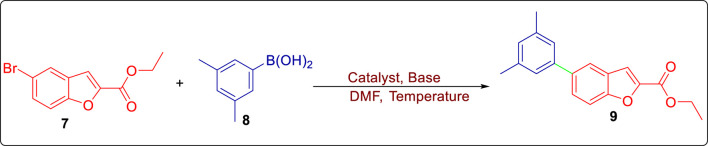					
Sr. no.	Catalyst	Base	Temperature (^o^C)	Time (h)	Yield (%)
**1**	Pd (acac)_2_	Aq. K_2_CO_3_ ^a^	80	6	10
**2**	Pd (acac)_2_	K_3_PO_4_ ^a^	90	6	8
**3**	Pd_2_ (dba)_3_	Aq. Na_2_CO_3_ ^a^	80	6	50
**4**	Pd_2_ (dba)_3_	Cs_2_CO_3_ ^a^	60	8	30
**5**	Pd_2_ (dba)_3_	Aq. Na_2_CO_3_ ^b^	80	4	50
**6**	Pd (dppf)Cl_2_	Aq. Na_2_CO_3_ ^b^	80	4	52
**7**	Pd (dppf)Cl_2_	Aq. Na2CO_3_ ^a^	25	5.5	48
**8**	Pd (dppf)Cl_2_	Aq. K_2_CO_3_ ^b^	80	4	55
**9**	Pd (dppf)Cl_2_	Aq. K2CO_3_ ^a^	25	6	52
**10**	PdCl_2_(PPh_3_)_2_	Aq. Na_2_CO_3_ ^a^	80	4	60
**11**	PdCl_2_(PPh_3_)_2_	Aq. K_2_CO_3_ ^a^	80	6	53
**12**	PdCl_2_(PPh_3_)_2_	Aq. Na_2_CO_3_ ^b^	80	2	67
**13**	Pd(PPh_3_)_4_	Aq. Na_2_CO_3_ ^b^	80	2	92
**14**	Pd(PPh_3_)_4_	Aq. K_2_CO_3_ ^b^	80	2	85

^a^ Reaction conditions: **7** (1 mmol), **8** (1 mmol), dimethylformamide (DMF), solvent, conventional heating/stirring.

^b^ Ultrasound-assisted protocol.

**SCHEME 2:**
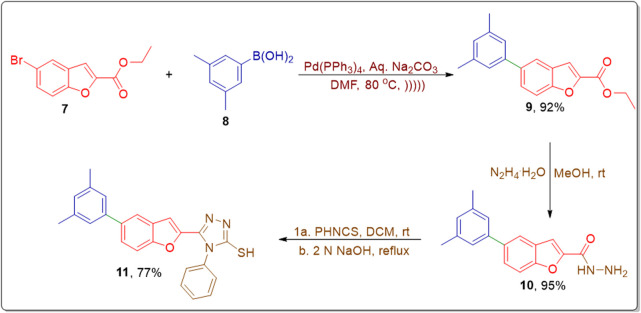
Utilization of optimized reaction conditions within the synthetic pathway to access the arylated benzofuran-triazole framework.

Under optimized conditions, the SMC reaction was carried out by treating aryl halide **7** with substituted aryl boronic acids **8** to attain coupled product **9** in an efficient yield of 92%. The obtained biaryl was further made to react with hydrazine monohydrate in methanol to afford the respective benzohydrazide **10** in a remarkable yield of 95%. The benzohydrazide then underwent reaction with phenylisothiocyanate in dichloromethane (DCM), followed by base-mediated cyclization to furnish the corresponding triazole framework **11** in a 77% yield (Scheme 2) (Zahoor et al., 2024b).

**SCHEME 3:**
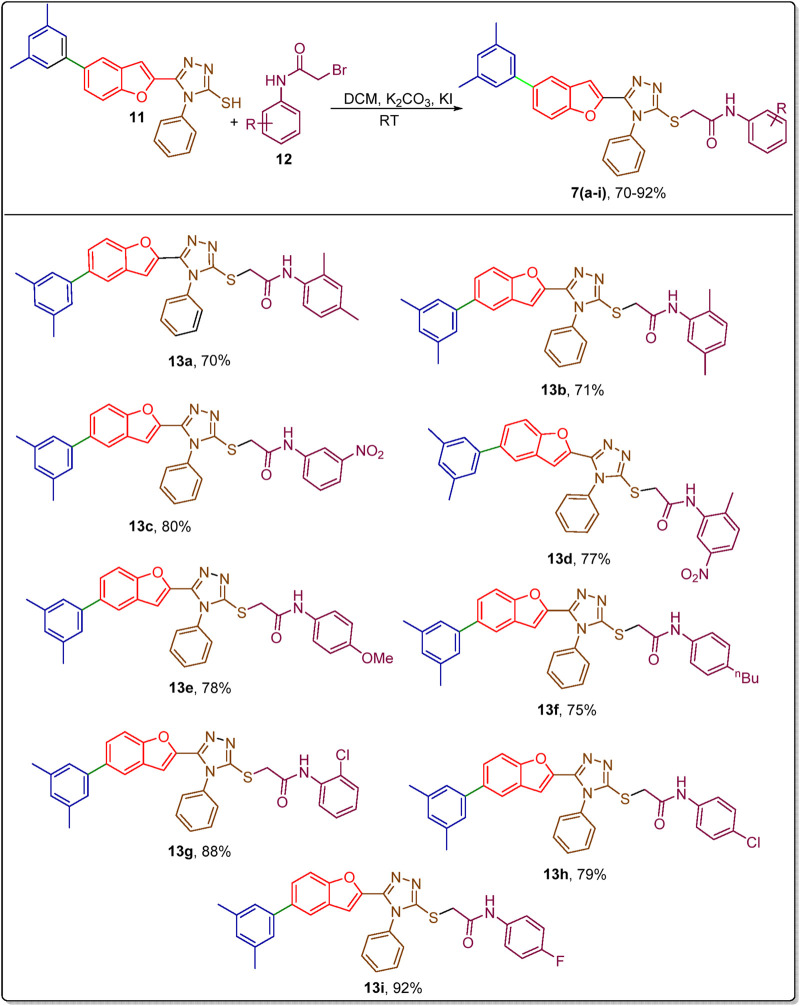
Substrate scope for the synthesis of arylated benzofuran-triazole hybrids **13(a–i)**.

The substituted phenylacetamides (prepared by treating substituted anilines with bromoacetyl bromide) (Irfan et al., 2022) were further treated with the benzofuran-based triazole framework **11** by employing potassium carbonate and potassium iodide in DCM. In this way, this synthetic strategy resulted in the generation of novel arylated benzofuran-triazole hybrids **13(a-i)** in a good to efficient yield range (72%–90%) (Scheme 3).

Diversely substituted phenyl acetamides were treated to determine the substrate scope of the synthetic protocol. All of the target molecules were obtained successfully. The analysis of synthesized compounds with their respective yields revealed the potential influence of electron-donating and electron-withdrawing groups on the yield of targeted derivatives. The electron-donating substituents (Me, OMe, and n-Bu) bearing derivatives **13a**, **13b**, **13e**, and **13f** were attained in 70%–78% yield. Meanwhile, the electron-withdrawing substituents (NO_2_, Cl, and F) bearing derivatives **13(c, g-i)** were attained in better yields (80%–92%). However, the 2-methyl 5-nitro substituted hybrid **13d** was achieved in 77% yield (Scheme 3).

All of the synthesized derivatives were structurally characterized by several spectroscopic techniques, including NMR spectroscopy (^1^H-NMR and ^13^C-NMR) and mass spectrometric analysis. In the ^1^H-NMR spectrum of the 2,4-dimethyl substituted derivative **13a**, the two singlets at 2.26 ppm and 2.34 ppm correspond to the methyl protons of **13a**. In addition, the singlet peak of two protons at 4.33 ppm arises as a result of -CH_2_ attached to the sulfur atom. The singlet at 6.71 ppm corresponds to the signal of furan-H. The aromatic protons gave signals in the range of 6.98–7.69 ppm. In addition, -NH gave a singlet signal at 9.55 ppm. For the ^13^C-NMR spectrum, the characteristic aliphatic carbon signals were found at 17.9 ppm, 21.1 ppm, 21.3 ppm, and 36.0 ppm. In addition, the carbon signal of the carbonyl carbon was observed at 166.5 ppm. Furthermore, an [M + H]^+^ peak was observed at 559.0 via mass spectrometry analysis, and the found value was significantly close to the theoretical value. The structural characterization of all compounds was carried out in a similar manner.

## Materials and methods

### Chemicals and instruments

All the requisite precursors, chemical reagents, and solvents were purchased from Macklin (China, Shanghai Pudong New Area) and Sigma-Aldrich (St. Louis, MO). These analytical-grade chemicals and solvents were used to carry out the synthetic protocol without additional purification. Thin-layer chromatography was carried out at each step to analyze the progress of the reaction. The melting points (mp) of synthesized compounds were obtained via a WRS-1B melting point apparatus, which have been used uncorrected. ^13^C-NMR spectra and ^1^H-NMR spectra of synthesized hybrids were obtained via a Bruker spectrophotometer run at 100 MHz/400 MHz and 400 MHz/600 MHZ, respectively. Deuterated chloroform and dimethyl sulfoxide were used for solubility, and tetramethyl silane (TMS) was employed as an internal standard. MestReNova software was used to interpret the spectral details.

### Synthesis of compounds **13 (a–i)**

The synthesized benzofuran-endowed aryl halide **7** (1 mmol) and substituted boronic acid **8** (1 mmol) were dissolved in DMF (5 mL). After 30 min of room temperature stirring, Pd(PPh_3_)_4_ (5 mol%) and 1 M Na_2_CO_3_ solution (5.5 mL) were added to the reaction mixture, which was subjected to ultrasonication for approximately 90–120 min. After indication of completion of reaction by thin layer chromatography, solvent extraction was performed with ethyl acetate and water, followed by drying the moisture content in the organic layer via anhydrous Na_2_SO_4_. Column chromatography (n-hexane:ethyl acetate 99:1) of the concentrated organic layer was then performed to obtain the pure product 9. The biaryl 9 was then used as precursor to synthesize the novel arylted benzofuran-triazole conjugates 13(a-i) via reported protocol (Zahoor et al., 2024b).

**13a**, White powder; 70%; mp 217–219 °C; ^1^H-NMR (400 MHz, CDCl_3_) 9.55 (s, 1H), 7.67 (d, *J* = 16 Hz, 5H), 7.54 (d, *J* = 8 Hz, 2H), 7.41 (d, *J* = 12 Hz, 2H), 7.14 (s, 2 H), 6.98 (s, 3H), 6.71 (s, 1H), 4.33 (s, 2H), 2.34 (s, 9H), 2.26 (s, 3H). ^13^C NMR (100 MHz, CDCl_3_) 166.5, 154.3, 140.8, 142.5, 141.7, 138.3, 138.3, 137.6, 136.1, 135.1, 132.7, 131.1, 130.4, 130.4, 130.4, 130.2, 128.8, 127.6, 127.3, 127.3, 127.3, 126.1, 125.7, 125.2, 125.2, 125.2, 122.9, 120.0, 111.7, 108.2, 36.0, 21.3, 21.1, 17.9. MS (m/z): 559.0 [M^+^ + H]. Elem. Anal. Calc. for C_34_H_30_N_4_O_2_S: C, 73.09; H, 5.41; N, 10.03. Found: C, 73.07; H, 5.39; N, 10.07.

**13b**, Off-white powder; 71%; mp 170–172 °C; ^1^H NMR (400 MHz, CDCl_3_) 9.61 (s, 1H), 7.76 (s, 1H), 7.63-7.61 (m, 1H), 7.53-7.51 (m, 2H), 7.45 (d, *J* = 8 Hz, 1H), 7.39 (d, *J* = 8 Hz, 3H), 7.14 (s, 2H), 7.03 (d, *J* = 8 Hz, 1H), 6.96 (s, 1H), 6.84 (d, *J* = 4 Hz, 2H), 6.50 (s, 1H), 4.09 (s, 2H), 2.34 (s, 9H), 2.29 (s, 3H). ^13^C NMR (100 MHz, CDCl_3_) 166.5, 154.3, 140.8, 138.3, 138.3, 137.6, 136.1, 132.7, 131.1, 130.4, 130.4, 130.4, 130.4, 130.2, 128.8, 127.3, 127.3, 127.3, 127.3, 126.1, 125.7, 125.2, 125.2, 125.2, 125.2, 122.9, 120.0, 111.7, 108.2, 36.0, 21.3, 21.1, 21.1, 17.9. MS (m/z): 559.0 [M^+^ + H]. Elem. Anal. Calc. for C_34_H_30_N_4_O_2_S: C, 73.09; H, 5.41; N, 10.03. Found: C, 73.07; H, 5.45; N, 10.05.

**13c**, White solid; 80%; mp 175–177 °C; ^1^H NMR (400 MHz, CDCl_3_) 10.99 (s, 1H), 8.67 (s, 1H), 7.89-7.83 (m, 2H), 7.66-7.60 (m, 5H), 7.51-7.49 (m, 1H), 7.41-7.38 (m, 4H), 7.09 (s, 2H), 6.93 (s, 1H), 4.15 (s, 2H), 2.30 (s, 6H). ^13^C NMR (100 MHz, DMSO) 166.7, 157.5, 155.5, 155.3, 154.4, 154.0, 152.7, 147.6, 147.3, 143.5, 143.0, 140.4, 138.3,138.3, 136.9, 130.7, 130.2, 129.6, 129.4, 129.0, 128.1, 128.1, 127.7, 125.2, 125.2, 120.4, 113.6, 111.9, 107.7, 55.3, 37.2, 21.4. MS (m/z): 576.0 [M^+^ + H]. Elem. Anal. Calc. for C_32_H_25_N_5_O_4_S: C, 66.77; H, 4.38; N, 12.17. Found: C, 66.79; H, 4.42; N, 12.19.

**13d**, Off-white solid; 77%; mp 156–158 °C; ^1^H NMR (400 MHz, CDCl_3_) 10.29 (s, 1H), 8.98 (s, 1H), 7.88 (d, *J* = 8 Hz, 1H), 7.43-7.66 (m, 8H), 7.30 (d, *J* = 8 Hz, 1H), 7.14 (s, 2H), 6.97 (s, 1H), 6.49 (s, 1H), 4.14 (s, 2H), 2.56 (s, 3H), 2.35 (s, 6H). ^13^C NMR (100 MHz, CDCl_3_) 166.8, 154.4, 148.3, 146.6, 142.1, 140.7, 138.3, 138.3, 137.7, 137.1, 136.2, 132.5, 131.4, 130.8, 130.5, 130.5, 130.5, 128.8, 127.5, 127.2, 126.4, 125.2, 125.2, 125.2, 120.1, 119.2, 116.7, 111.7, 108.6, 36.12, 21.3, 21.3, 19.0. MS (m/z): 589.9 [M^+^]. Elem. Anal. Calc. for C_33_H_27_N_5_O_4_S: C, 67.22; H, 4.62; N, 11.88. Found: C, 67.28; H, 4.66; N, 11.86.

**13e**, White powder; 78%; mp 152–154 °C; ^1^H NMR (400 MHz, CDCl_3_) 10.13 (s, 1H), 7.67-7.59 (m, 5H), 7.54 (t, *J* = 8 Hz, 3H), 7.46 (d, *J* = 8 Hz, 1H), 7.39 (d, *J* = 8 Hz, 2H), 7.15 (s, 1H), 6.97 (s, 1H), 6.83 (d, *J* = 8 Hz, 2H), 6.43 (s, 1H), 3.97 (s, 2H), 3.76 (s, 3H), 2.34 (s, 6H). ^13^C NMR (100 MHz, CDCl_3_) 166.0, 165.9, 156.3, 154.4, 154.3, 142.7, 140.9, 138.3, 137.6, 132.8, 131.4, 131.2, 130.4, 130.4, 129.2, 128.7, 127.6, 127.3, 127.2, 126.1, 125.2, 124.3, 121.3, 120.0, 114.0, 113.2, 111.7, 107.9, 106.8, 55.4, 36.0, 21.3, 21.3. MS (m/z): 561.0 [M^+^ + H]. Elem. Anal. Calc. for C_33_H_28_N_4_O_3_S: C, 70.69; H, 5.03; N, 9.99. Found: C, 70.71; H, 5.07; N, 9.97.

**13f**, White solid; 75%; mp 191–193 °C; ^1^H NMR (400 MHz, CDCl_3_) 10.19 (s, 1H), 7.62-7.38 (m, 10H), 7.15-7.09 (m, 4H), 6.96 (s, 1H), 6.44 (s, 1H), 3.98 (s, 2H), 2.54 (t, *J* = 8 Hz, 2H), 2.34 (s, 6 H), 1.56-1.24 (m, 4H), 0.89 (t, *J* = 6 Hz, 3H). ^13^C NMR (100 MHz, CDCl_3_) 166.1, 154.4, 148.2, 142.7, 140.9, 138.9, 138.3, 138.3, 137.6, 135.8, 132.8, 131.1, 130.4, 130.4, 128.7, 128.7, 127.6, 127.3, 127.3, 126.1, 125.2, 125.2, 125.2, 120.0, 119.7, 119.7, 119.6, 111.7, 107.9, 36.1, 35.0, 33.6, 22.2, 21.3, 21.3, 13.9. MS (m/z): 587.0 [M^+^ + H]. Elem. Anal. Calc. for C_36_H_34_N_4_O_2_S: C, 73.69; H, 5.84; N, 9.55. Found: C, 73.73; H, 5.86; N, 9.57.

**13g**, Off-white powder; 88%; mp 216–218 °C; ^1^H NMR (400 MHz, CDCl_3_) 9.87 (s, 1H), 8.30 (d, *J* = 12 Hz, 1H), 7.34-7.62 (m, 10H), 7.14 (s, 2H), 7.02 (t, *J* = 8 Hz, 1H), 6.96 (s, 1H), 6.50 (s, 1H), 4.12 (s, 2H), 2.34 (s, 6H). ^13^C NMR (100 MHz, CDCl_3_) 154.3, 153.4, 148.4, 142.9, 140.9, 138.2, 138.2, 137.5, 134.9, 132.9, 131.0, 130.3, 130.3, 129.3, 128.7, 127.6, 127.3, 127.3, 127.3, 127.3, 127.3, 125.9, 125.2, 125.2, 125.0, 124.1, 122.2, 119.9, 111.7, 107.8, 35.8, 21.3. MS (m/z): 564.9 [M^+^]. Elem. Anal. Calc. for C_32_H_25_ClN_4_O_24_S: C, 68.02; H, 4.46; N, 9.91. Found: C, 68.06; H, 4.42; N, 9.95.

**13h**, Off-white powder; 79%; mp 224–226 °C; ^1^H NMR (400 MHz, CDCl_3_) 10.49 (s, 1H), 7.67-7.59 (m, 7H), 7.55-7.47 (m, 2H), 7.39 (d, *J* = 8 Hz, 2H), 7.27 (s, 1H), 7.15 (s, 2H), 6.97 (s, 1H), 6.44 (s, 1H), 3.96 (s, 2H), 2.34 (s, 6 H). ^13^C NMR (100 MHz, CDCl_3_) 166.4, 154.4, 151.2, 150.5, 150.2, 149.4, 148.8, 148.8, 142.5, 140.8, 138.3, 137.6, 136.8, 135.8, 134.2, 132.7, 131.2, 130.4, 128.9, 128.8, 127.2, 126.2, 125.2, 120.9, 120.0, 111.7, 110.1, 110.1, 108.0, 36.0, 21.3, 21.3. MS (m/z): 564.9 [M^+^]. Elem. Anal. Calc. for C_32_H_25_ClN_4_O_2_S: C, 68.02; H, 4.46; N, 9.91. Found: C, 68.04; H, 4.48; N, 9.95.

**13i**, Off-White solid; 92%; mp 253–255 °C; ^1^H NMR (400 MHz, CDCl_3_) 10.45 (s, 1H), 7.67-7.61 (m, 6H), 7.54 (d, *J* = 8, 1H), 7.47 (d, *J* = 8 Hz, 1H), 7.40 (d, *J* = 4 Hz, 2H), 7.15 (s, 2 H), 7.01-6.97 (m, 3H), 6.47 (s, 1H), 4.01 (s, 2H), 2.34 (s, 6H). ^13^C NMR (100 MHz, CDCl_3_) 166.1, 160.5, 154.4, 140.8, 138.3, 138.3, 138.3, 137.7, 134.2, 132.6, 131.3, 130.4, 130.4, 130.4, 128.8, 127.5, 127.2, 127.2, 126.3, 125.2, 125.2, 125.2, 121.4, 121.3, 120.1, 115.6, 115.3, 111.7, 108.3, 36.1, 21.3, 21.3. MS (m/z): 549.0 [M^+^ + H]. Elem. Anal. Calc. for C_32_H_25_FN_4_O_2_S: C, 70.06; H, 4.59; N, 10.21. Found: C, 70.04; H, 4.57; N, 10.19%.

## Conclusion

To conclude, we have developed a facile, additive-free, and convenient protocol for the Suzuki cross-coupling reaction to synthesize substituted biaryls in high yields. The reaction was carried out by treating benzofuran-endowed aryl halide and substituted boronic acids using aq. Na_2_CO_3_ and Pd(PPh_3_)_4_ under ultrasonic conditions to access the coupling products within a minimum time. Compared to previous studies, the developed protocol has been found to be facile and efficient in terms of reaction conditions, catalyst loading, and product yields, as it does not require an inert atmosphere or the use of additives. The obtained products were efficiently employed as synthetic building blocks to synthesize a novel series of arylated benzofuran-based phenyl acetamides. The merits of the utilized methodology are the facile conversion, short duration, broad substrate scope, non-inert atmosphere, and high yields.

## Data Availability

The original contributions presented in the study are included in the article/Supplementary Material; further inquiries can be directed to the corresponding authors.
