# Supplementary material for: Ultrasonic-assisted, additive-free Pd-catalyzed Suzuki–Miyaura cross-coupling enabled synthesis of novel arylated benzofuran-triazole hybrids
Source: Front Chem. 2025 Dec 10;13:1726528. doi: 10.3389/fchem.2025.1726528 (PMC12728044; doi:10.3389/fchem.2025.1726528)
Supplement: Supplementary file 1 [file DataSheet1.docx]

**Ultrasonic-assisted, additive-free Pd-catalyzed Suzuki-Miyaura cross-coupling enabled synthesis of novel arylated benzofuran-triazole hybrids**

Muhammad Jawwad Saif^1^, Sajjad Ahmad^2^, Aqsa Mushtaq^3^, Saba Munawar^3^, Ameer Fawad Zahoor^3,*^, Shoela Ettampola^4^, Ali Irfan^3^, Katarzyna Kotwica-Mojzych^5^, Karol Ruszel^6^, Mariusz Mojzych^6,*^

^1^Department of Applied Chemistry, Government College University Faisalabad, Faisalabad-38000, Pakistan.

^2^Department of Chemistry, University of Engineering and Technology Lahore, Faisalabad Campus, Faisalabad-38000, Pakistan.

^3^Department of Chemistry, Government College University Faisalabad, Faisalabad-38000, Pakistan.

^4^Dunwoody High School, 5035 Vermack Rd, Dunwoody, GA 30338, USA.

^5^Department of Histology, Embryology and Cytophysiology, Medical University of Lublin, Lublin, Poland

^6^Collegium Medicum, The Mazovian Academy in Płock, Pl. Dąbrowskiego 2, 09-402 Płock, Poland.

*Correspondence: [fawad.zahoor@gcuf.edu.pk](mailto:fawad.zahoor@gcuf.edu.pk); [m.mojzych@mazowiecka.edu.pl](mailto:m.mojzych@mazowiecka.edu.pl)

**Table of Contents**

1. **NMR characterization of synthesized hybrids**

**Figure S 1.1:** ^1^H-NMR Spectra of compound **13a**.

**Figure S 1.2:** ^13^C-NMR Spectra of compound **13a**.

**Figure S 1.3:** ^1^H-NMR Spectra of compound **13b**.

**Figure S 1.4:** ^13^C-NMR Spectra of compound **13b**.

**Figure S 1.5:** ^1^H-NMR Spectra of compound **13c**.

**Figure S 1.6:** ^13^C-NMR Spectra of compound **13c**.

**Figure S 1.7:** ^1^H-NMR Spectra of compound **13d**.

**Figure S 1.8:** ^13^C-NMR Spectra of compound **13d**.

**Figure S 1.9:** ^1^H-NMR Spectra of compound **13e**.

**Figure S 1.10:** ^13^C-NMR Spectra of compound **13e**.

**Figure S 1.11:** ^1^H-NMR Spectra of compound **13f**.

**Figure S 1.12:** ^13^C-NMR Spectra of compound **13f**.

**Figure S 1.13:** ^1^H-NMR Spectra of compound **13g**.

**Figure S 1.14:** ^13^C-NMR Spectra of compound **13g**.

**Figure S 1.15:** ^1^H-NMR Spectra of compound **13h**.

**Figure S 1.16:** ^13^C-NMR Spectra of compound **13h**.

**Figure S 1.17:** ^1^H-NMR Spectra of compound **13i**.

**Figure S 1.18:** ^13^C-NMR Spectra of compound **13i.**

**NMR Spectra**


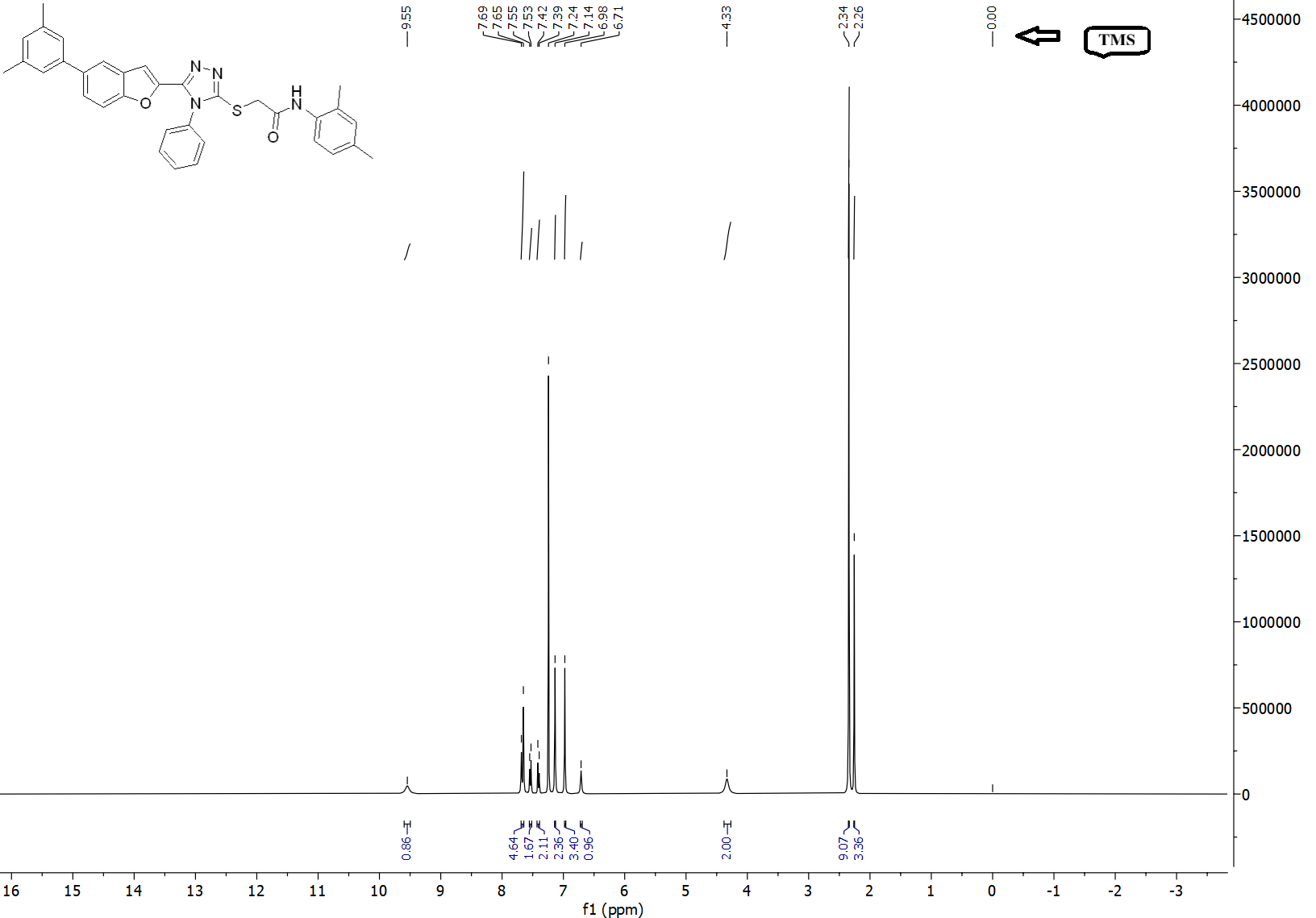


**Figure S 1.1:** ^1^H-NMR Spectra of compound **13a**.

**
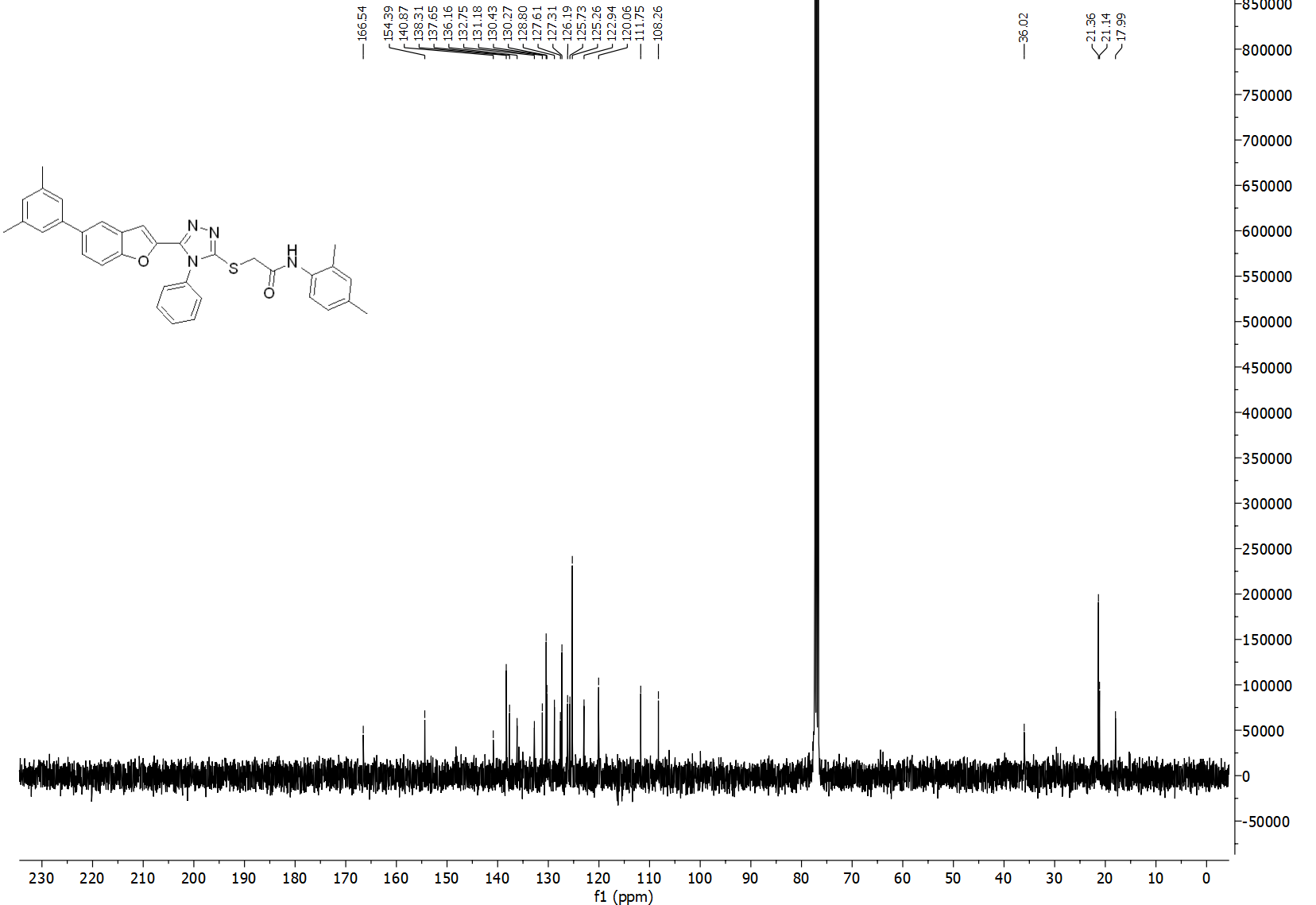
**

**Figure S 1.2:** ^13^C-NMR Spectra of compound **13a**.


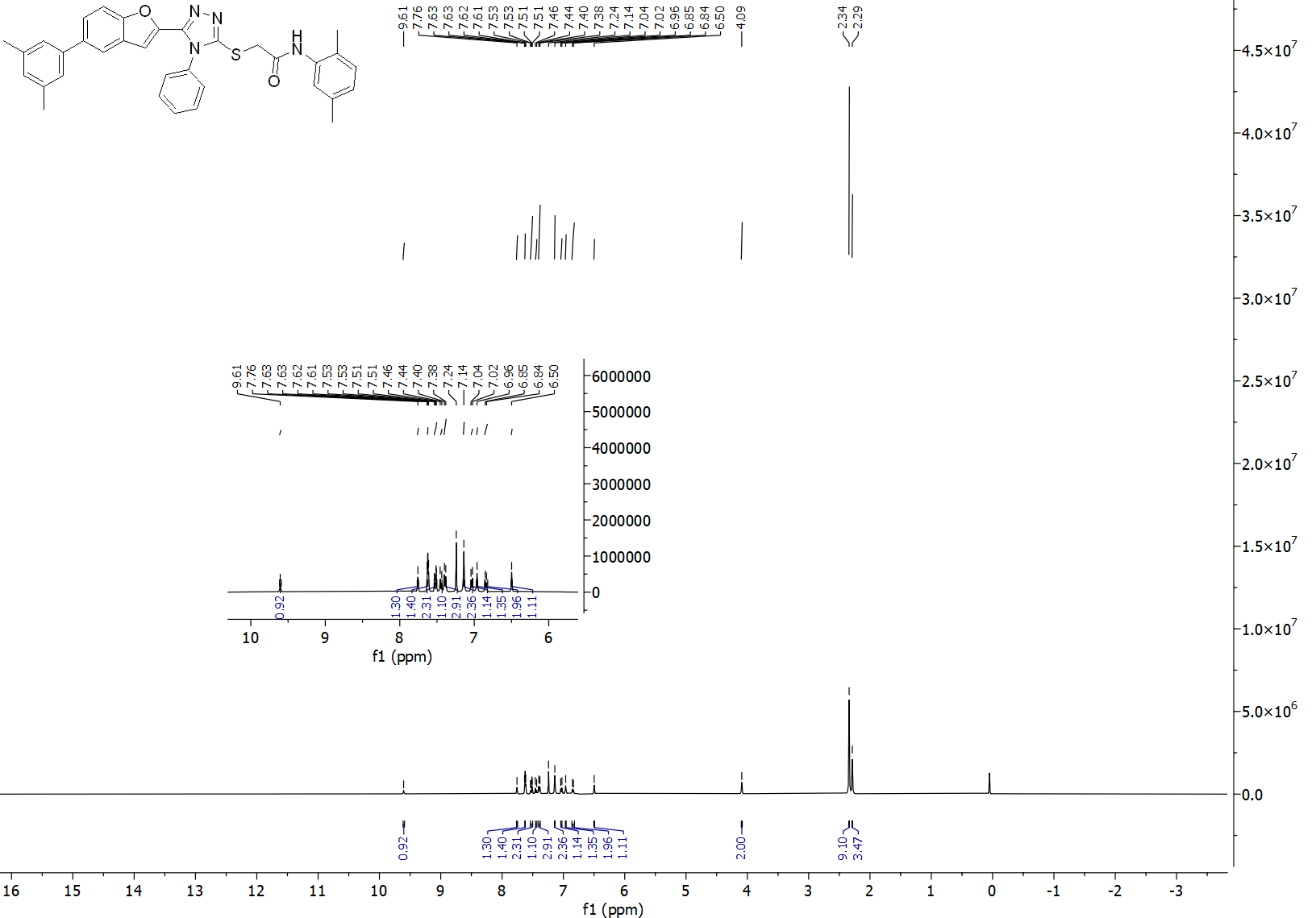


**Figure S 1.3:** ^1^H-NMR Spectra of compound **13b**.

**
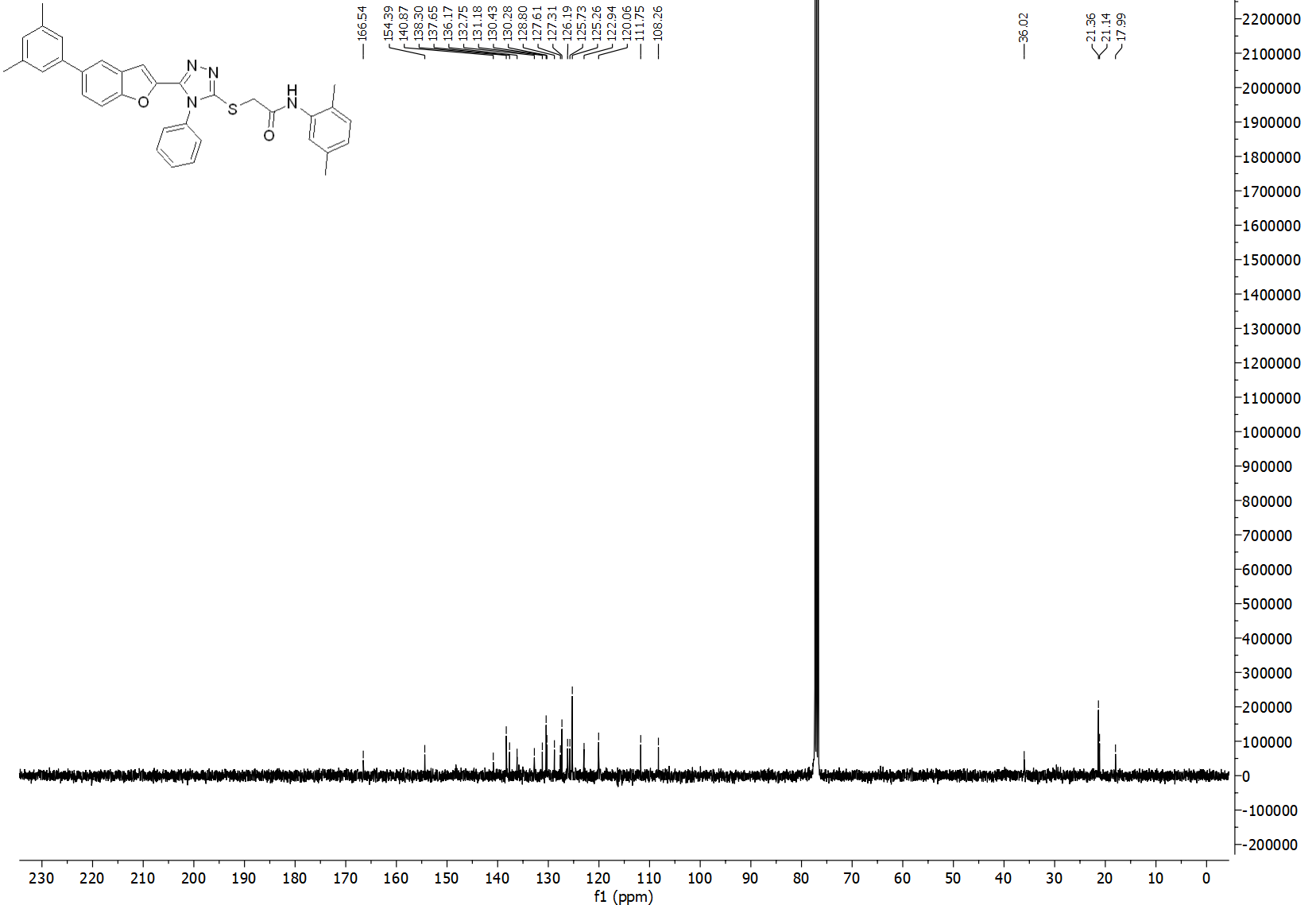
**

**Figure S 1.4:** ^13^C-NMR Spectra of compound **13b**.


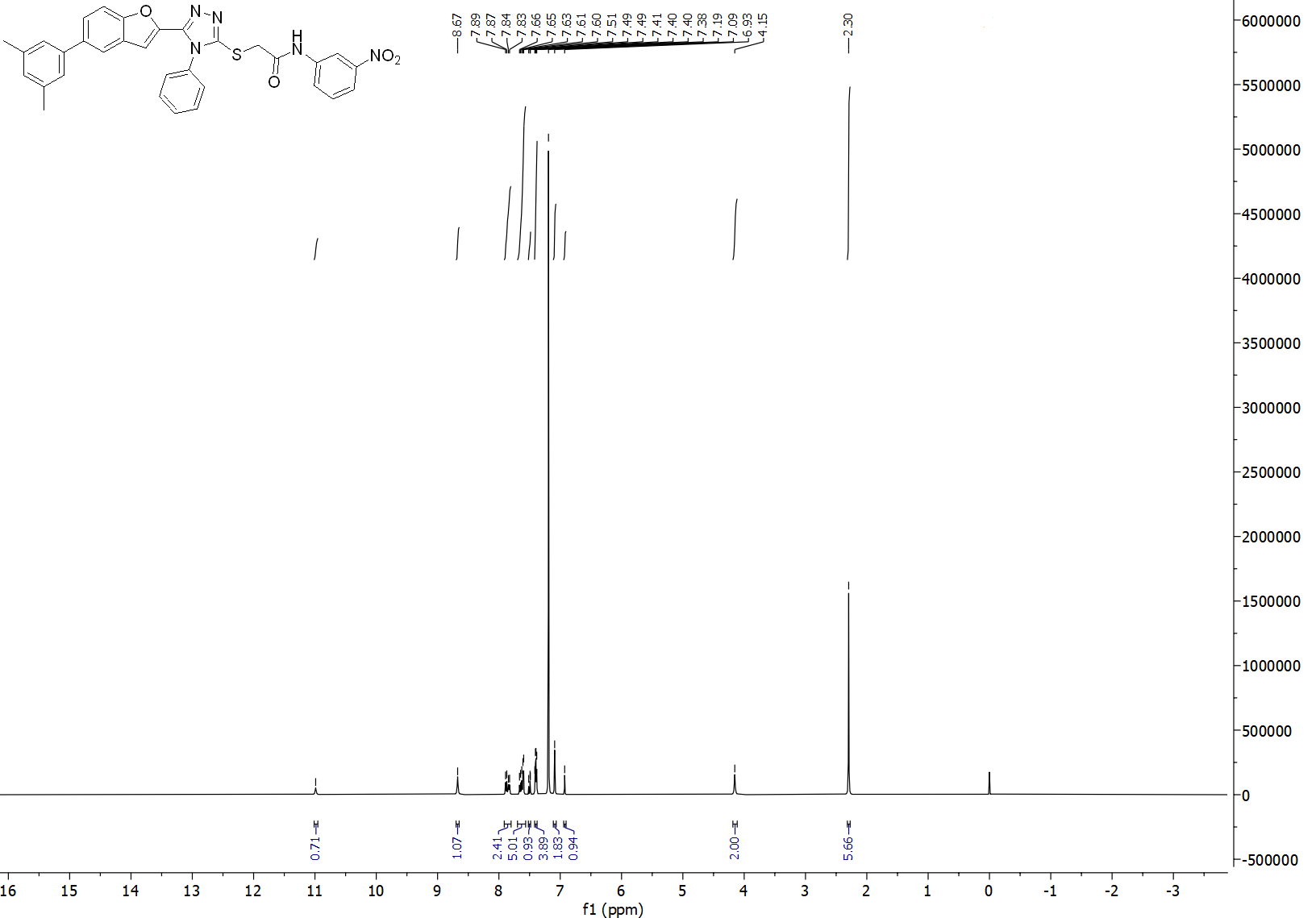


**Figure S 1.5:** ^1^H-NMR Spectra of compound **13c**.


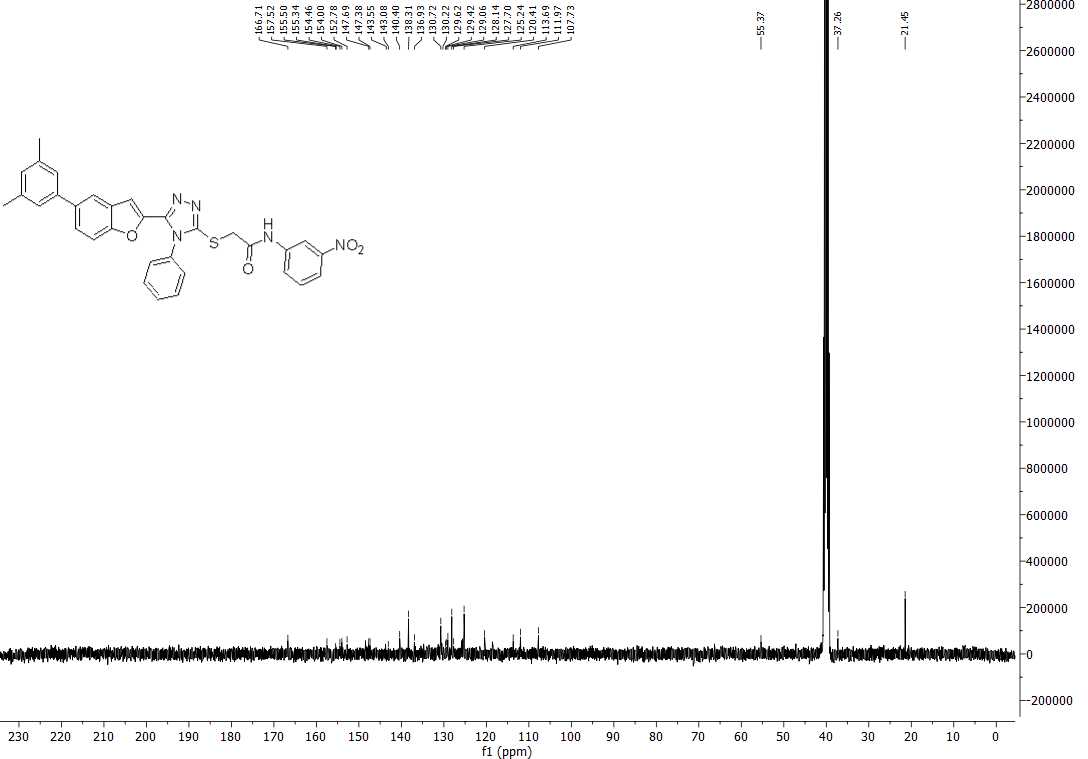
 **Figure S 1.6:** ^13^C-NMR Spectra of compound **13c**.


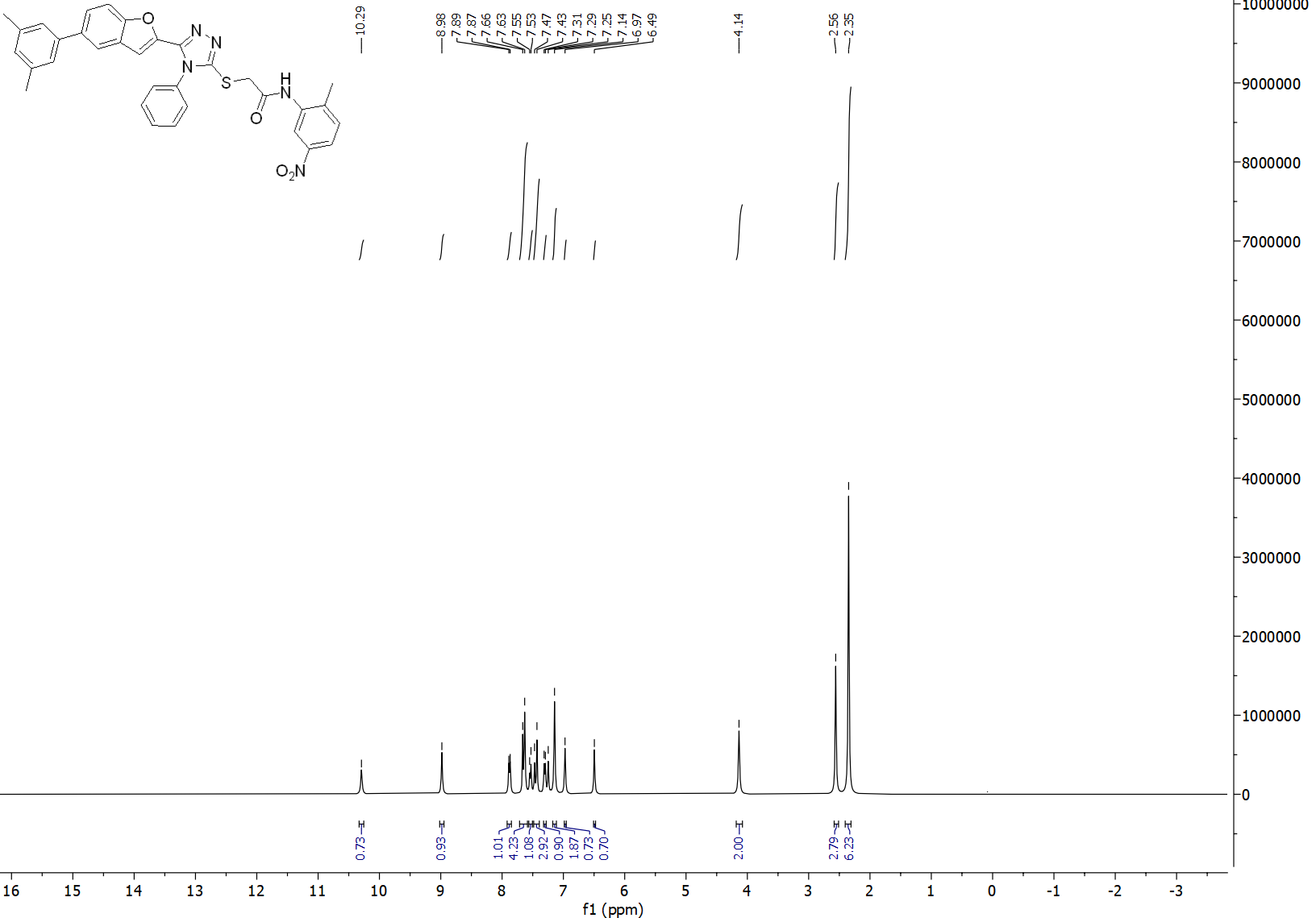


**Figure S 1.7:** ^1^H-NMR Spectra of compound **13d**.

**
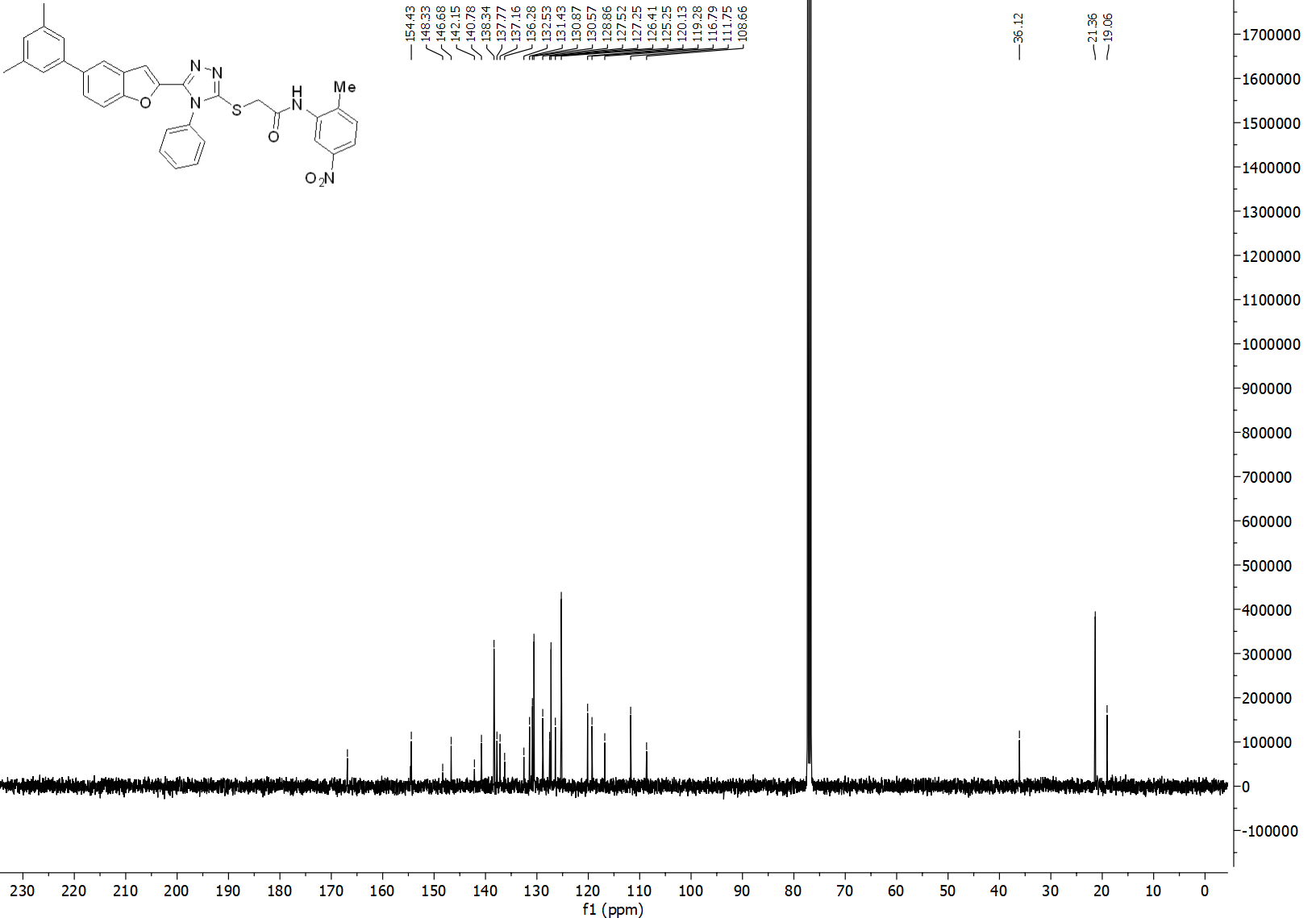
**

**Figure S 1.8:** ^13^C-NMR Spectra of compound **13d**.


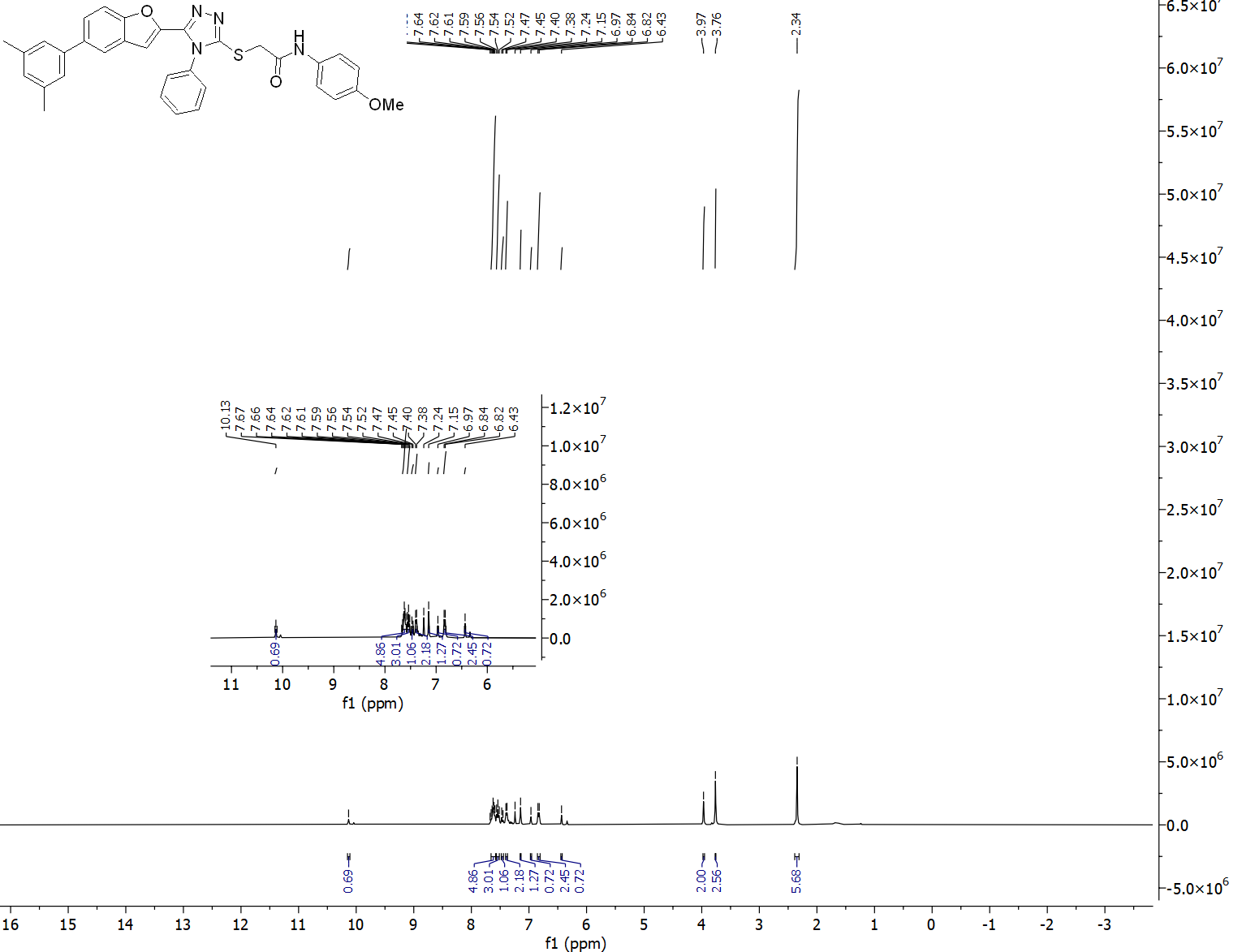


**Figure S 1.9:** ^1^H-NMR Spectra of compound **13e**.

**
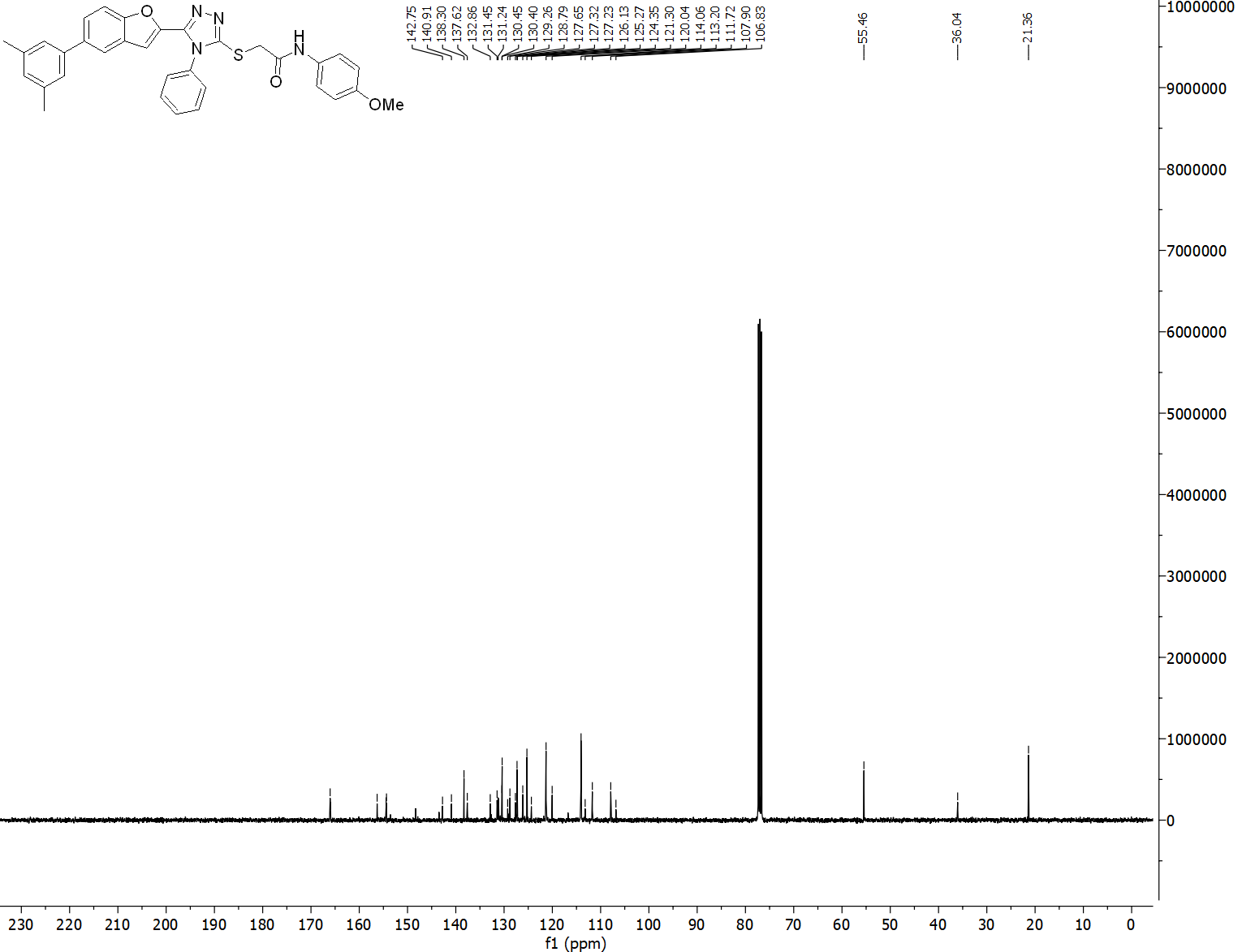
**

**Figure S 1.10:** ^13^C-NMR Spectra of compound **13e**.


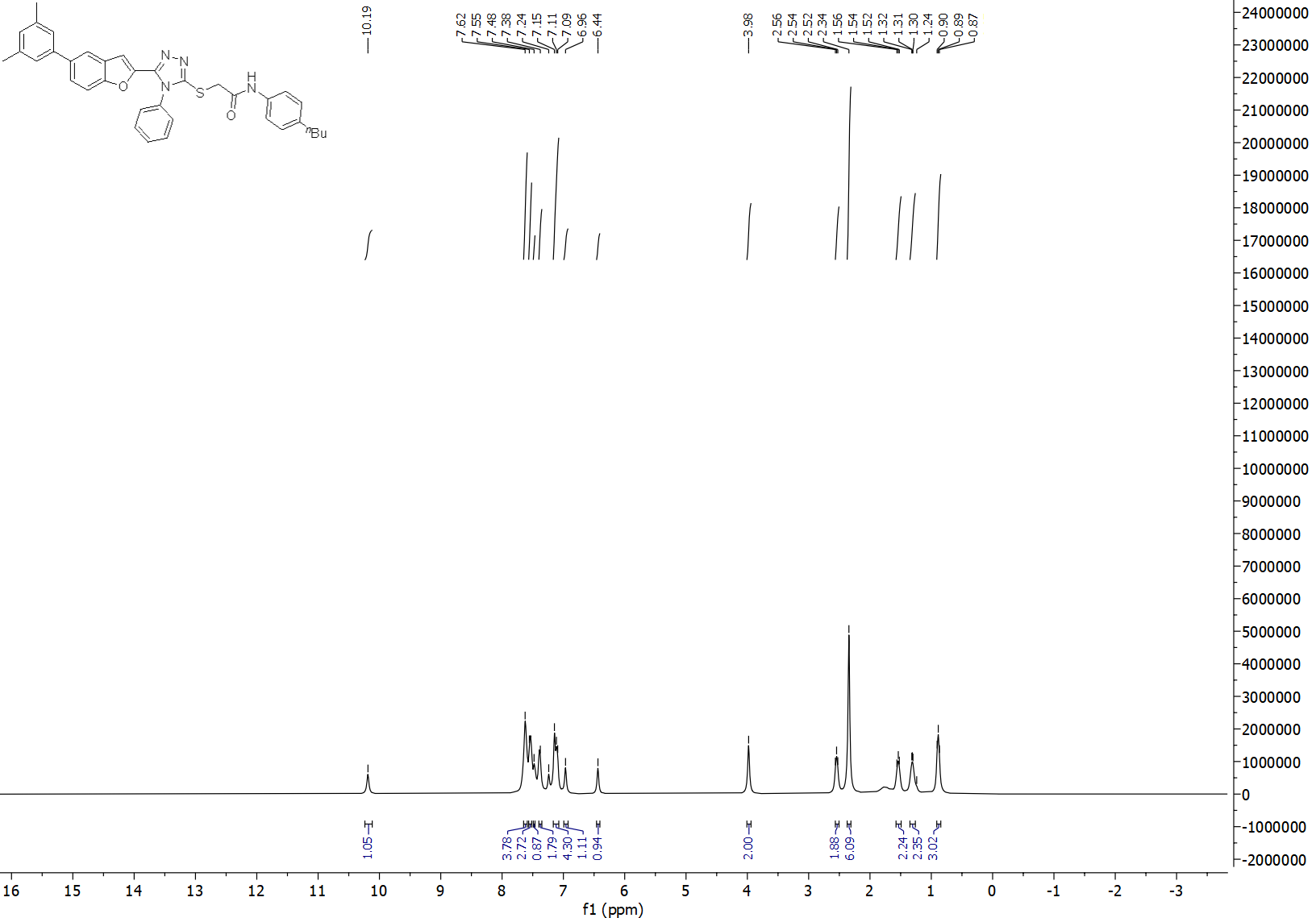


**Figure S 1.11:** ^1^H-NMR Spectra of compound **13f**.

**
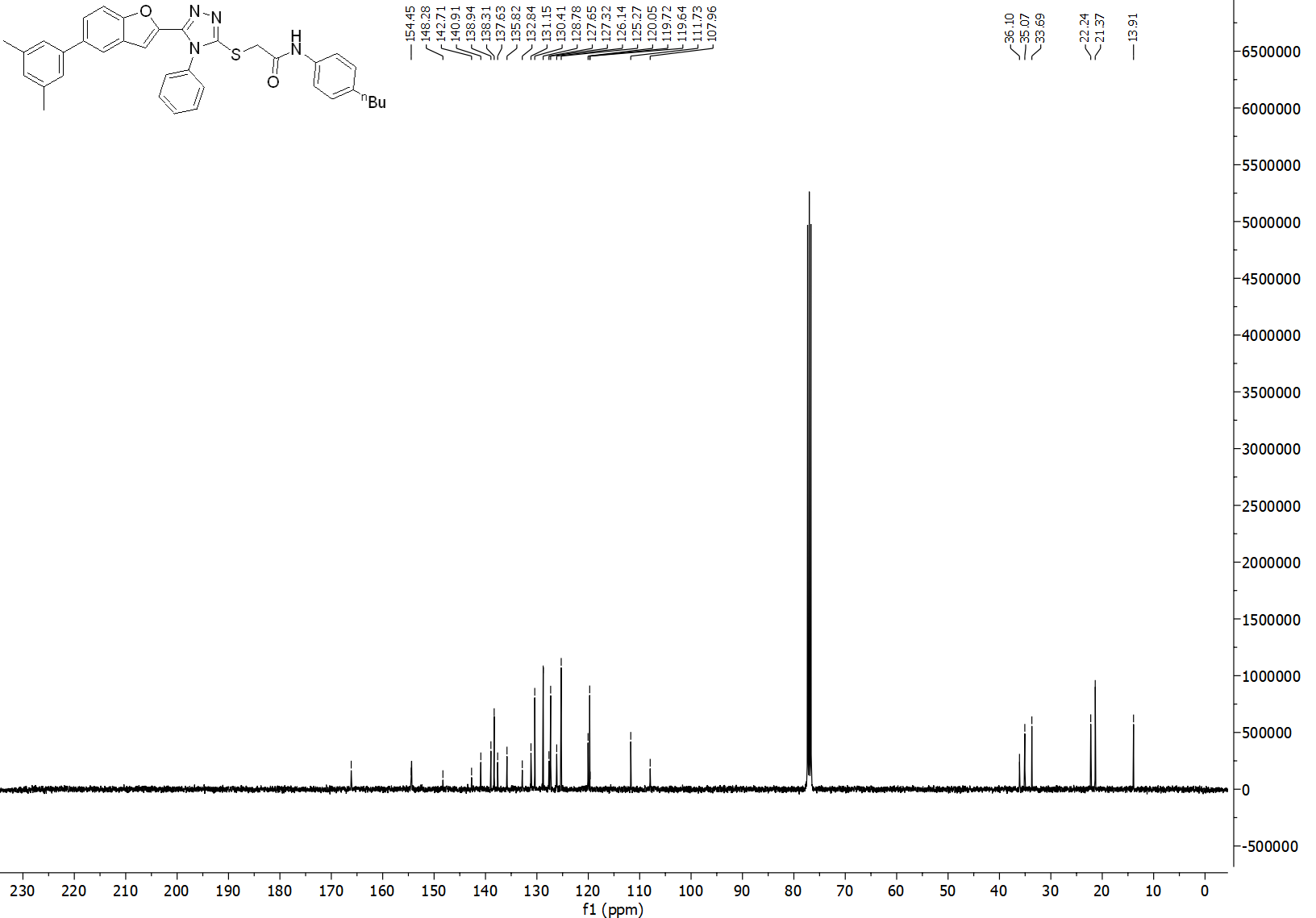
**

**Figure S 1.12:** ^13^C-NMR Spectra of compound **13f**.


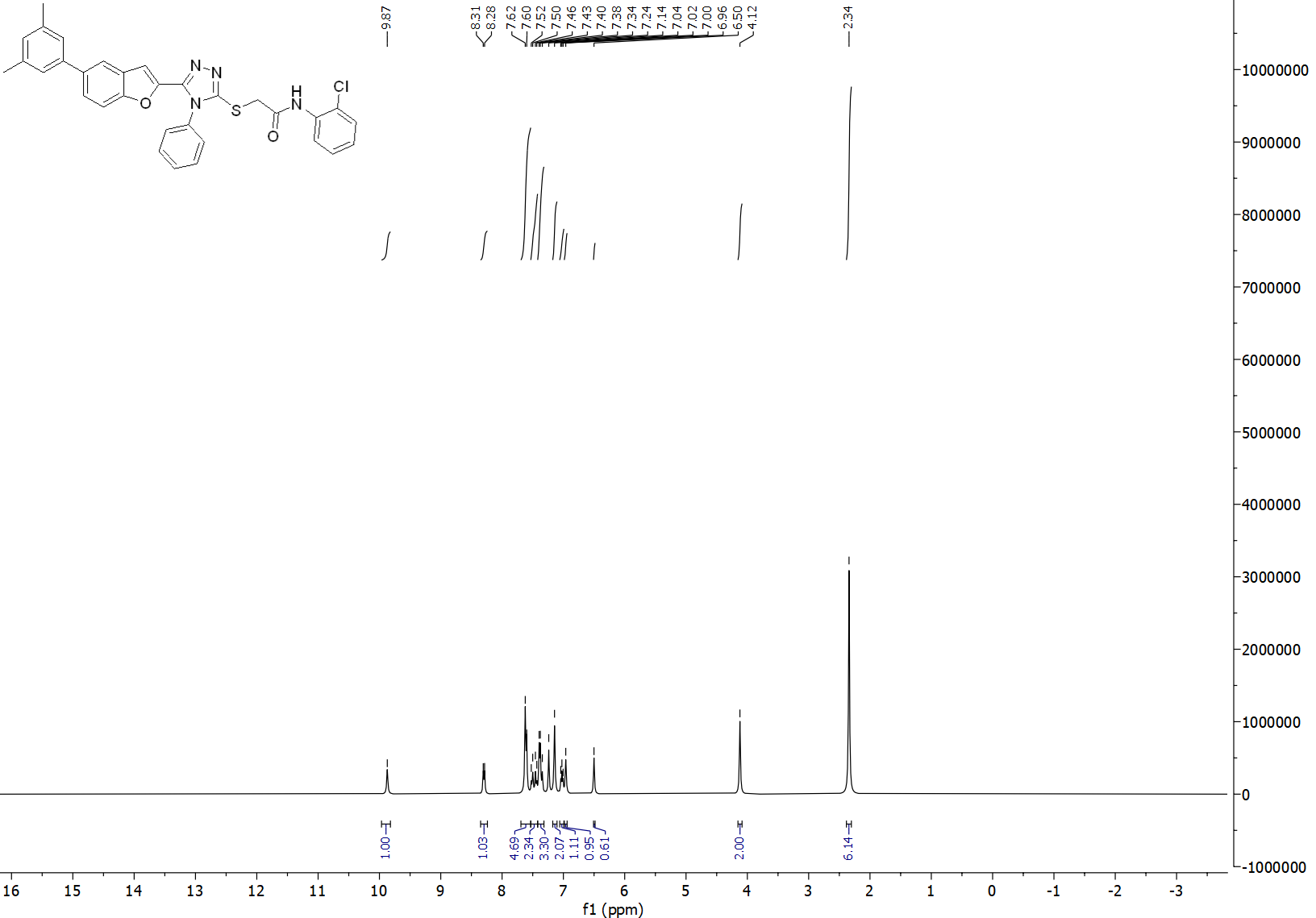


**Figure S 1.13:** ^1^H-NMR Spectra of compound **13g**.

**
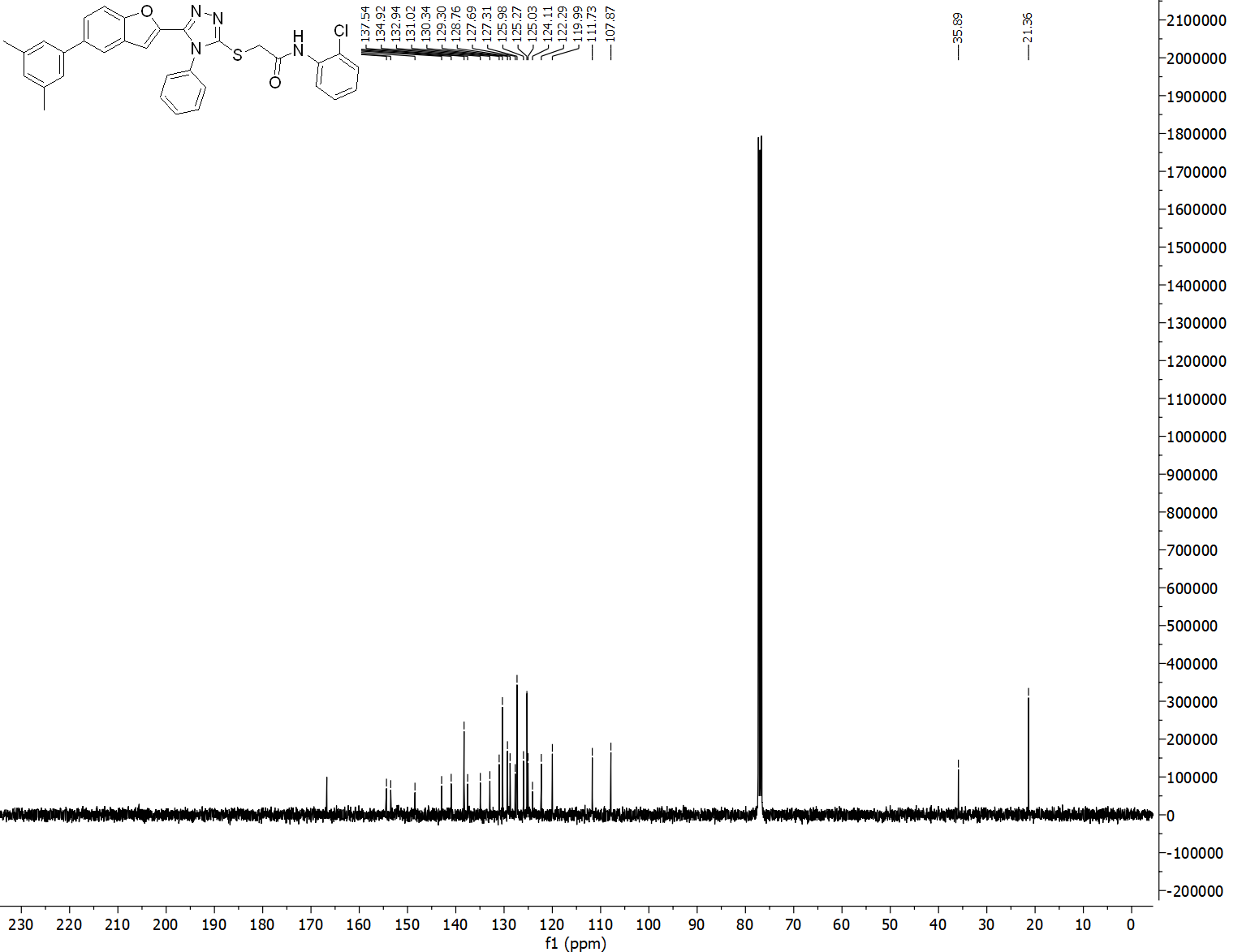
**

**Figure S 1.14:** ^13^C-NMR Spectra of compound **13g**.


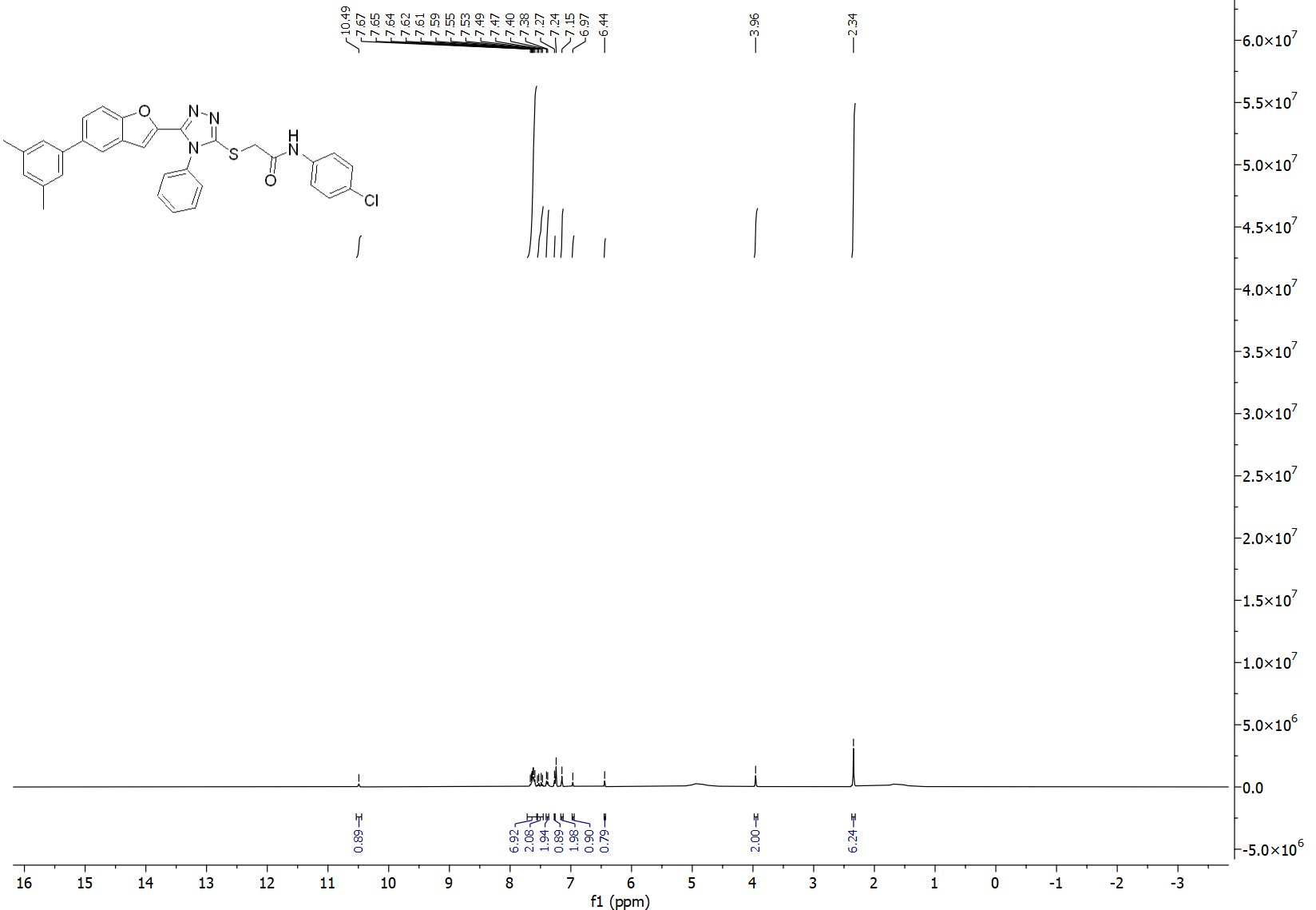


**Figure S 1.15:** ^1^H-NMR Spectra of compound **13h**.


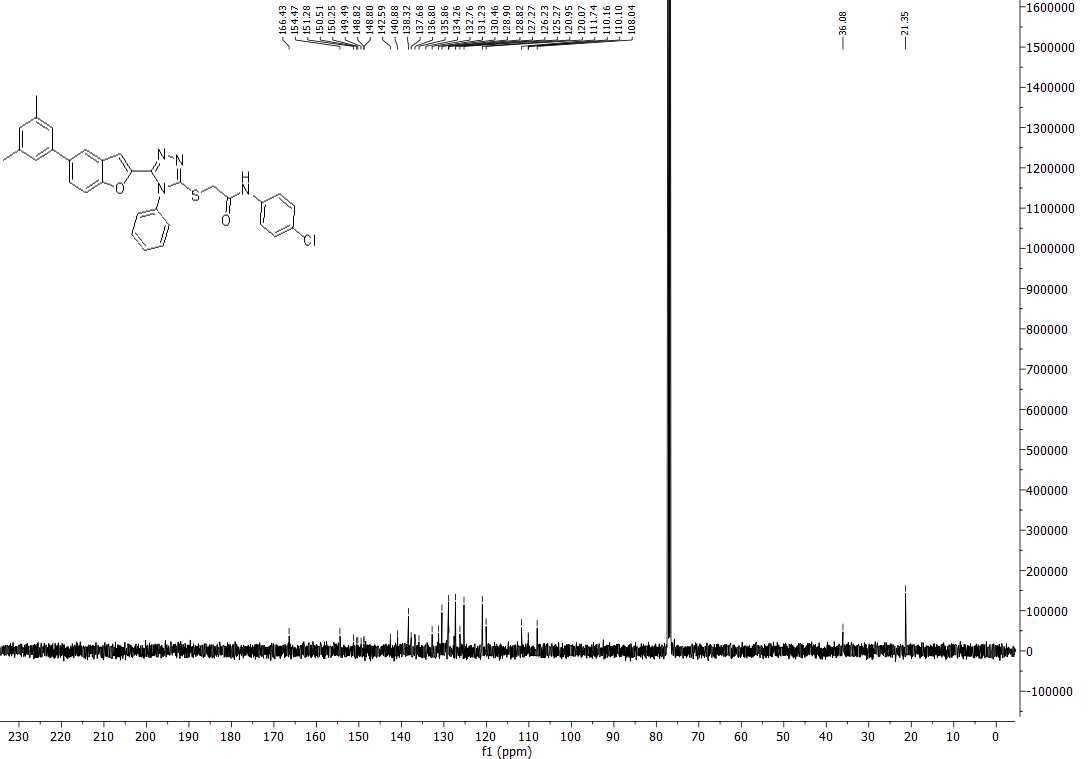


**Figure S 1.16:** ^13^C-NMR Spectra of compound **13h**.


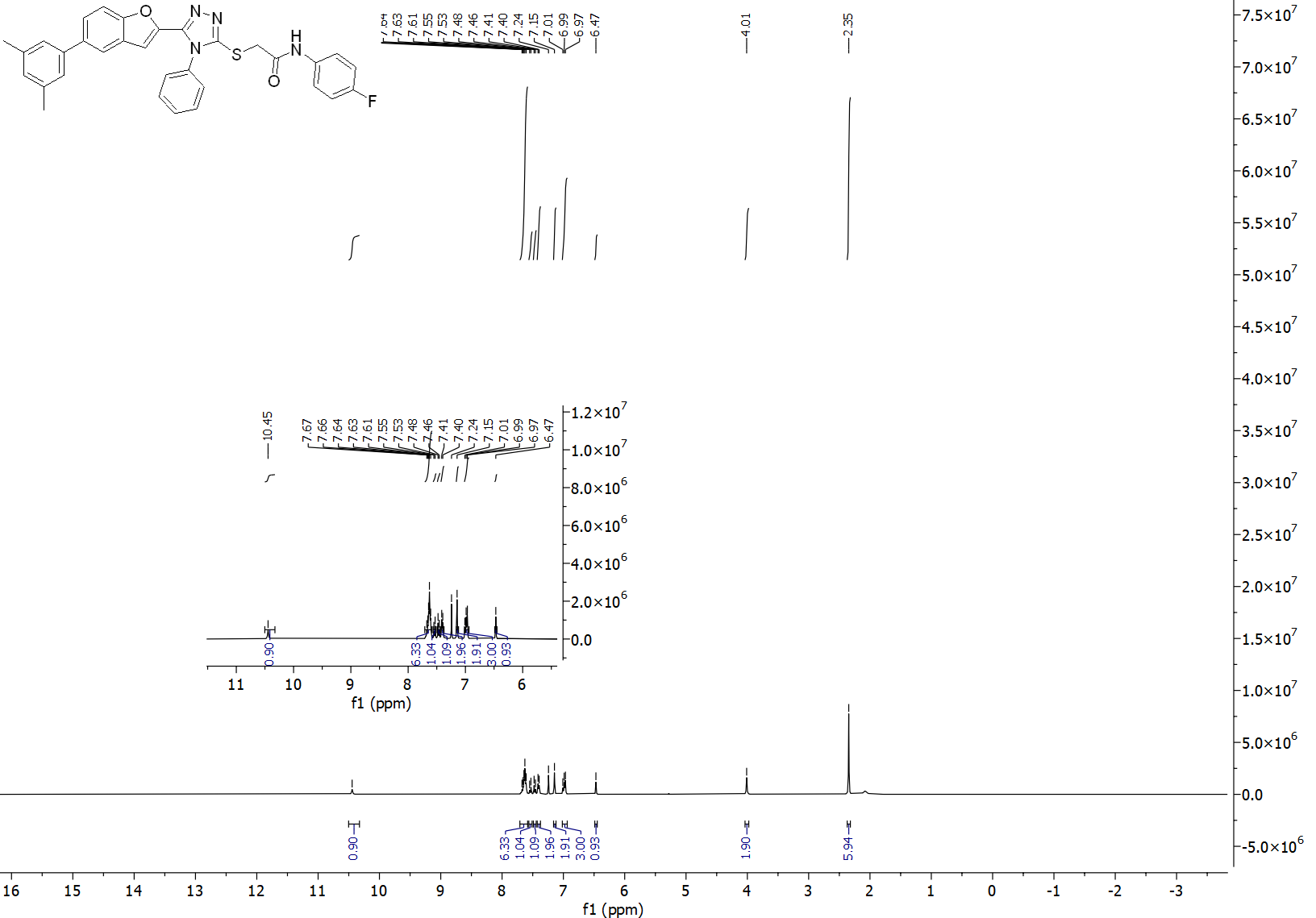


**Figure S 1.17:** ^1^H-NMR Spectra of compound **13i**.


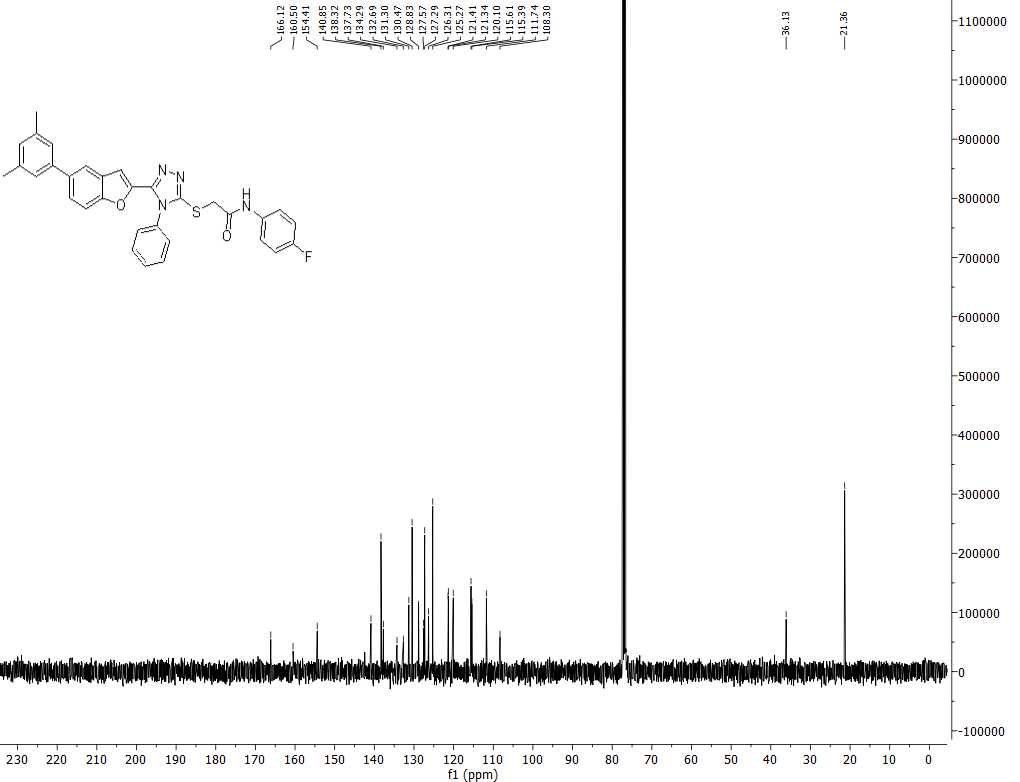


**Figure S 1.18:** ^13^C-NMR Spectra of compound **13i**.
